# Comparison Results of Three-Port Robot-Assisted and Uniportal Video-Assisted Lobectomy for Functional Recovery Index in the Treatment of Early Stage Non-small Cell Lung Cancer: A Propensity Score-Matched Analysis

**DOI:** 10.1245/s10434-023-14767-8

**Published:** 2023-12-17

**Authors:** Haixiao Diao, Lin Xu, Xiao Li, Yancheng Wang, Zhongmin Peng

**Affiliations:** 1 Shandong Provincial Hospital, Shandong University, Jinan, China; 2 Shandong Provincial Hospital Affiliated to Shandong First Medical University, Jinan, China; 3 Shandong Provincial Hospital, Shandong First Medical University, Jinan, China

**Keywords:** Robot-assisted thoracic surgery, Uniportal video-assisted thoracic surgery, Postoperative rehabilitation, Propensity score-matched analysis

## Abstract

**Background:**

Minimally invasive lobectomy is the standard treatment for early stage non-small cell lung cancer (NSCLC). The aim of this study is to investigate postoperative recovery in a prospective trial of discharged patients with early stage non-small cell lung cancer undergoing robot-assisted thoracic surgery (RATS) versus uniportal video-assisted thoracic surgery (UVATS).

**Patients and Methods:**

This is a prospective and observational study. From 9 September 2022 to 1 July 2023, 178 patients diagnosed with NSCLC admitted to the Department of Thoracic Surgery of Shandong Provincial Hospital signed informed consent and underwent lobectomy by RATS and UVATS. The functional recovery index included MD Anderson Symptom Inventory, Christensen Fatigue Scale, EORTC QLQ-C30, and Leicester Cough Questionnaire.

**Results:**

After propensity score-matched analysis, each group included 42 cases. For the baseline characteristics of patients, operation time (*p* = 0.01) and length of stay (*p* = 0.04) were shorter in the RATS group. The number of lymph nodes resected in the RATS group was much more than in the UVATS group. According to our investigation, appetite loss, nausea, diarrhea, and cough severity after RATS were better than after UVATS. After the first week, pain severity degree of the RATS group was higher than UVATS, while there was no difference during the second and third week. The physical score of the RATS group was higher than the UVATS group (*p* = 0.04), according to the Leicester Cough Questionnaire.

**Conclusion:**

RATS was associated with severe short-term postoperative pain but less postoperative complications.

Lung cancer was the leading cause of cancer death in 2020, according to GLOBOCAN. There were 2.2 million new cancer cases and 1.8 million deaths in 2020, which is about one in ten diagnosed advanced cancer cases (11.4%) and one in five deaths (18.0%).^1^ The 5-year survival rate of patients with diagnosed lung cancer is only 10–20%.^2^ However, owing to development of high-resolution computed tomography (CT), more early stage non-small cell lung cancers (NSCLCs) were identified. In these patients, lobectomy remains the gold-standard curative treatment method.^3,4^

With the progress of thoracoscopic technology, minimally invasive surgery (MIS) has become a well-established treatment for early stage NSCLC.^5^ Robot-assisted and uniportal video-assisted thoracoscopic surgery are the two most widely used MIS modalities. Compared with uniportal video-assisted thoracic surgery (UVATS), robot-assisted thoracic surgery (RATS) provides an amplified, high-definition, three-dimensional (3D) visualization of the surgical field, and the flexible robotic arms help the surgeon complete complex operations. However, UVATS had fewer surgical incisions. In a randomized clinical trial, Jin et al.^6^ reported that RATS and UVATS had comparable perioperative outcomes, but the RATS group had a significantly higher number of lymph nodes harvested than UVATS. However, the influence of the two modalities on postdischarge rehabilitation and delayed complications are still unknown.

This study aims to investigate the postoperative recovery of discharged patients from RATS and UVATS, such as activity, pain, exhaustion, sleep quality, and cough severity. Postoperative recovery is a complex process involving multiple dimensions, both physical and psychological, even at the social level.^7^ We adopted portable digital equipment to monitor patients’ activity, providing objective data. We also sent questionnaires to obtain fatigue scores, Numerical Rating Scale of pain, and cough degree scores.^8^ Overall, we compare and evaluate the after-discharge patients who underwent RATS and UVATS in multiple dimensions, such as the psychological, physical, and social.

## Patients and Methods

We carried out this prospective, observational study at the thoracic surgery department of Shandong Provincial Hospital affiliated with Shandong First Medical University. Every participant received an informed consent form and was informed of the details of the study during the preoperative conversation. This study was also approved by the Ethics Committee on Biomedical Research of Shandong Provincial Hospital.

We prospectively included patients with suspected or diagnosed clinical stage I, stage II, and stage IIIA non-small cell lung cancer (NSCLC) from 9 September 2022 to 1 July 2023. All patients were Chinese. The patients who underwent RATS or UVATS lobectomy were recruited in our study.

In our hospital, a thoracoscope can be used to complete stage I and stage II without lymph node metastasis NSCLC lobectomy or segmentectomy; however, complex operations such as sleeve lobectomy are difficult, and there is a high possibility of conversion to open thoracotomy. Generally, some conditions such as thoracic dense adhesion, bronchial invasion, chest wall invasion, hilar-dense nodal invasion, previous chemotherapy, and previous thoracic surgery are no longer regarded as absolute contraindications.

Additionally, the inclusion criteria were as follows: (1) patients aged between 18 and 80 years, (2) patients who underwent RATS or UVATS lobectomy, (3) patients with pathology proven positive for NSCLC, and (4) patients with well-tolerated cardiopulmonary function. The exclusion criteria were as follows: (1) patients who underwent segmentectomy, sleeve lobectomy, or wedge resection; (2) patients with limited mobility (such as those who use wheelchairs or require assistance when walking) or unhealthy mental conditions (including anxiety, depression, schizophrenia, and other psychological disorders); (3) patients with a history of multiple lung surgeries; (4) patients with previous neoadjuvant therapy; (5) patients with pathology proven positive for small cell lung cancer; (6) patients with a history of malignancy; or (7) follow-up data that could not be obtained.

### Preoperative Preparation

Patients in the study had 2–3 days to finish all necessary preoperative examinations, including to assess cardiopulmonary function (pulmonary function test, electrocardiographic, cardiac ultrasonography, and coronary CT performed if necessary). To determine the tumor stage, abdomen and adrenal ultrasound, chest CT, and brain CT were performed. These examinations ensured safety of surgery and excluded metastasis of cancer. In addition, Enhanced Recovery After Surgery (ERAS) was implemented throughout treatment in our thoracic surgery department.^9^

### Surgical Technique and Postoperative Hospital Stay

The skin incision made in UVATS was located between the midaxillary line and anterior axillary line of the fourth or fifth intercostal space and was approximately 4 cm long. The machine used during RATS was da Vinci Si (Intuitive Surgical, Sunnyvale, California). We adopted a three-arm approach, which is a slightly modified version described by Dylewski.^10^ We incised a 4-cm incision in the fifth intercostal space on the anterior axillary line as an assistant utility port and first instrument port, then incised a 1-cm incision in the eighth intercostal space on the midaxillary line as a lens port. A 1-cm incision made in the eighth intercostal space behind the posterior axillary line served as the second instrument port. A distance of 8 cm was kept between each port. The lymph node approach was identical in RATS and UVATS and was done in accordance with National Comprehensive Cancer Network (NCCN) guidelines. Generally, patients with NSCLC should receive N1 and N2 nodule resection and at least three mediastinal lymph node station samplings or a LN dissection (left: 4L, 5L, 6L, 7L, 8L, 9L groups; right: 2R, 4R, 7R, 8R, 9R groups).^11^ One drainage tube (24FR) was placed in all patients. The drainage tube of UVATS patients was placed dorsal of incision, while the drainage tube of RATS patients was placed through the lens port.

All patients were treated with low-molecular-weight heparin to prevent thrombus after surgery, unless the drainage fluid was bright red in color. Postoperative analgesia was provided by non-steroidal anti-inflammatory drugs (NSAIDs). Opioids were used if the effect of the NSAIDs was unsatisfactory. The afternoon of the day after surgery, a DR scan was arranged for all patients to exclude atelectasis and pleural effusion. Patients were discharged on the second day or third day once the chest drain was removed and no complications occurred.

In addition, the baseline characteristics and perioperative outcomes were recorded, including: age, gender, body mass index (BMI), smoking history, pulmonary function [maximal voluntary ventilation (MVV), forced expiratory volume in 1 s (FEV1), and diffusing capacity of the lung for CO (DLCO-SB)], American Society of Anesthesiologists-physical status (ASA-PS), Charlson Comorbidity index,^12^ operation time, surgical site, lymph node dissection number, length of hospital stay, postoperative drainage time, volume of drainage, perioperative complications, reintervention rate, reoperation rate, and mortality. The length of hospitalization was calculated by the number of postoperative hospitalization nights.

### Postoperative Follow-Up Parameters after Discharge

For follow-up, all questionnaires were obtained by WeChat, which is a chat tool in China. Patients are generally reviewed at around the fourth week after discharge; in total, we collected 3 weeks of after-surgery patient data. The questionnaires were sent to patients every week (on day 7, day 14, and day 21). If the questionnaire could not be returned within 1 day, the patient would be reminded, and samples longer than 2 days were discarded.

For daily activity and sleep time, we used smart bands (Mi Band 6,^13,14^ Xiaomi Corporation, Beijing, China) to monitor. They are unobtrusive, waterproof wristwear devices with a light sensor and 1-week battery life that automatically records daily activity and sleep time. They can record daily steps, 1 day of total sleep time, and heart rate.

For our study, all the rating scales are shown below.

### Numerical Rating Scale (NRS)^15^

The Numerical Rating Scale is one of the most commonly used pain scales in medicine. We used this scale to assess the degree of postoperative pain in patients. The NRS consists of a digital version and a visual analogue scale. Due to the limitations of our questionnaire, this study only has the digital version. Patients chose the score according to their own pain degree, and the higher the score, the higher the pain degree.

### Christensen Fatigue Scale (CFS)^16^

The purpose of the Christensen Fatigue Scale was to assess patient fatigue in our study. The scale evaluated fatigue on a scale of 1–10. Patients reporting a higher score (10) means they feel more fatigued. This scale is a single, self-rated scale that has been used in multiple clinical studies and is applicable to most questionnaires due to its simplicity. The fatigue scale model was used in this study to study the pathogenesis and treatment of postoperative fatigue syndrome.

### EORTC QLQ-C30^17^

The EORTC QLQ-C30 is a comprehensive assessment of the quality of life (QOL) of patients with cancer from multiple dimensions, such as physical, psychological, and social functioning. The validated and relevant questionnaire, the EORTC QLQ-C30, is one of the most widely used quality-of-life questionnaires in cancer research.^18^ The EORTC QLQ-C30 has the 15 following items: global health status (health status of the whole body), function subscales (physical functioning, role functioning, emotional functioning, cognitive functioning, and social functioning) and symptom subscales/items (fatigue, pain, nausea and vomiting, dyspnea, appetite loss, insomnia, constipation, diarrhea, and financial impact of the disease). A high score for a functional scale represents a high/healthy level of functioning; a high score for the global health status represents a high QOL, but a high score for a symptom scale represents a high level of symptomatology. The analysis of the questionnaire scores was performed according to the EORTC QLQ-C30 scoring manual.

### MD Anderson Symptom Inventory^19^

The MD Anderson Symptom Inventory scale is designed to capture the severity of the patient’s symptoms, as well as the extent to which these symptoms interfere with daily life, and this scale is used in the follow-up investigation of various cancers. The MD Anderson Symptom Inventory has the following items: pain, fatigue, nausea, disturbed sleep, distress/feeling upset, shortness of breath, difficulty remembering, lack of appetite, drowsiness, dry mouth, sadness, vomiting, numbness/tingling, walking, activity, working (including housework), relations with other people, and enjoyment of life mood. Higher scores indicate more severe symptoms. Other studies have shown that a rating of 5 or greater (on a 0–10 numeric rating scale) indicates a moderate-to-severe symptom that significantly impairs daily functioning.^20^

### Outcomes

The outcomes were the difference of the perioperative outcomes and the short-term (3-week) post-discharge index between the RATS group and the UVATS group to determine which surgical method is superior.

### Statistical Analysis

We analyzed the factors of the patients as comprehensively as possible. To mitigate the impact of nonrandom patient allocation and control for confounding variables, we employed a propensity score-matching (PSM) analysis including age, gender, BMI, MVV, FEV1, DLCO-SB, smoking status, Charlson Comorbidity Index, and length of the tumor. Match tolerance was 0.02. All analyses were performed with SPSS 26.0 (IBM-SPSS Inc, Armonk, NY). All discrepancies in both outcomes were tested using a *t*-test when expressing normal distribution. The median [interquartile range (IQR)] and rank sum test were utilized for non-normally distributed data. A chi-square test was used for dichotomous variables. Two-sided *p*-value < 0.05 was considered statistically significant. Visio 2021 (Microsoft, USA) was used to draw a flow diagram.

## Results

### Patient Enrollment and Questionnaire Collection

**Fig. 1:**
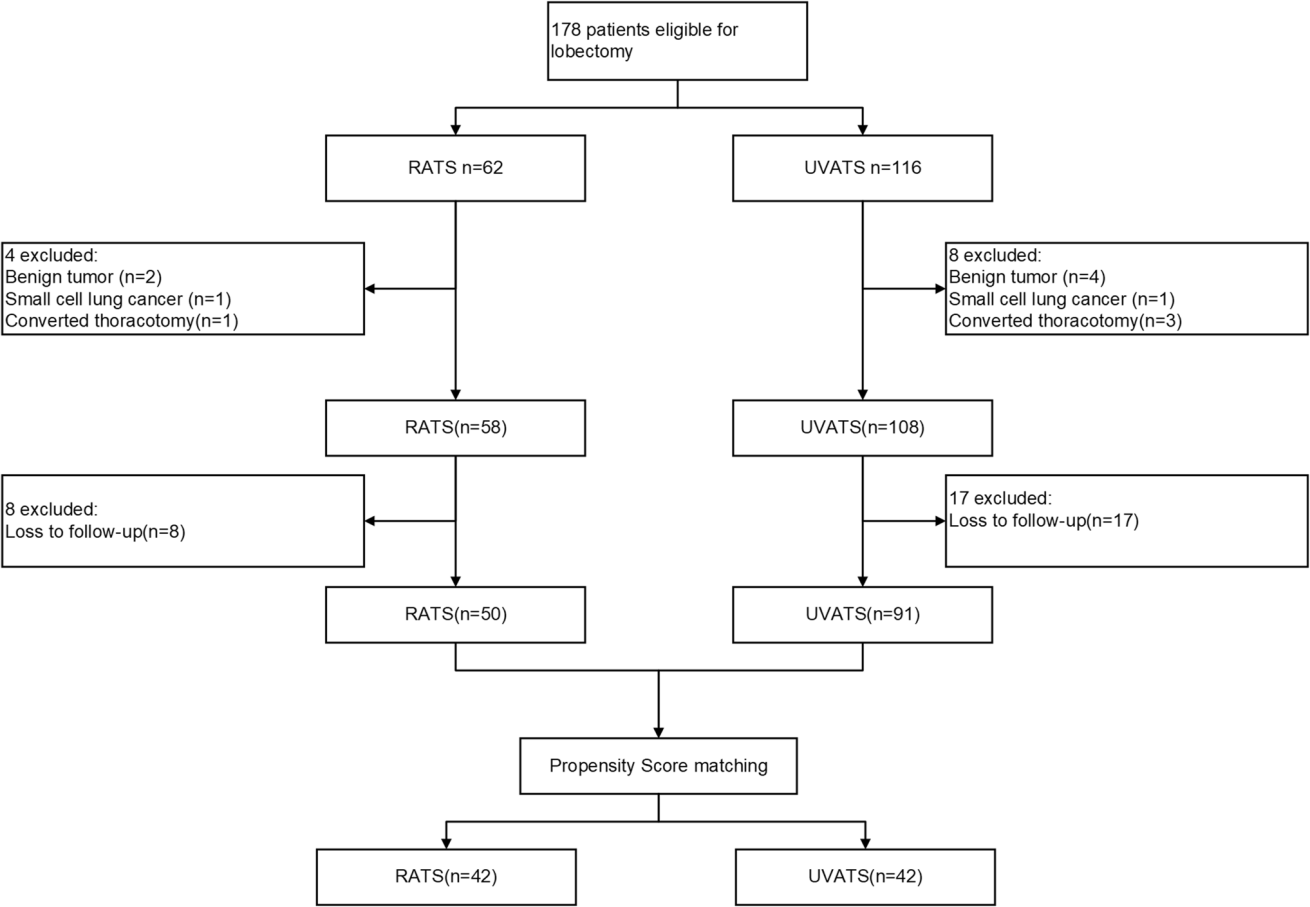
RATS, Robot-assisted thoracic surgery; UVATS, uniportal Video-Assisted Thoracic Surgery

As shown in Fig. 1, from 9 September 2022 to 1 July 2023, 178 patients were eligible, with a total of 62 patients who underwent RATS and 116 patients who underwent UVATS, and 12 excluded. Among those 12, 6 had benign tumors, 2 had small cell lung cancers, and 4 had converted to thoracotomies during operation. We sent questionnaires to all of the 166 enrolled patients. However, only 141 patients finished questionnaires and were available for further analysis. Finally, 50 patients who underwent RATS and 91 patients who underwent UVATS were included. After PSM, 42 patients in each group were well matched by a 1:1 PSM algorithm.

### Baseline Characteristics and Perioperative Outcomes

Patient baseline characteristics are listed in Table 1. Operation time [107 (90–121.25) min vs. 120 (103.75–130) min; *p* = 0.01] and length of stay [3 (2–3) days vs. 3 (3–4) days; *p* = 0.04] of the RATS group were shorter than the UVATS group. Lymphadenectomy in the RATS group was more than the UVATS group (*p* = 0.04). Age (*p* = 0.65), BMI (*p* = 0.53), gender (male %, *p* = 1.00), smoking status (*p* = 0.80), FEV1 (*p* = 0.95), MVV (*p* = 0.59), DLCO-SB (*p* = 0.82), Charlson Comorbidity Index (*p* = 0.34), ASA physical status (*p* = 0.24), solid nodules proportion (*p* = 0.50), tumor pathology (*p* = 0.09), length of tumor (*p* = 0.18), duration of chest drainage (days), and total drainage volume (*p* = 0.74) had no statistical difference between the RATS and UVATS groups.

**Table 1:** Baseline characteristics

Characteristics	All patients			Propensity score-matched patients		
	RATS (*n* = 50)	UVATS (*n* = 91)	*p*-value	RATS (*n* = 42)	UVATS (*n* = 42)	*p*-value
Age (years)	56.6	58.03	0.21	57.89	57.05	0.65
BMI (kg/m^2^)	24.98	25.21	0.71	25.02	24.59	0.53
Gender (male %)	22 (44%)	32 (35%)	0.44	18 (43%)	18 (43%)	1.00
Smoking status	11 (22%)	23 (25.2%)	0.47	11 (26%)	10 (23%)	0.80
FEV1 (%)	97.39%	99.90%	0.33	98.19%	97.96%	0.95
MVV (%)	92%	90.37%	0.59	91.64%	89.40%	0.59
DLCO-SB (%)	90.00%	90.60%	0.57	87.37%	88.20%	0.82
Charlson Comorbidity Index	2.775	2.573	0.45	2.79	2.5	0.34
ASA physical status			0.39			0.24
1–2	47 (94%)	83 (93%)		40 (95%)	37 (88%)	
3–4	3 (6%)	7 (7%)		2 (5%)	5(12%)	
Operation time (min, IQR)	110 (90–122.5)	120 (110–137.5)	0.01	107 (90–121.25)	120 (103.75–130)	0.01
Total drainage volume(ml, IQR)	350(255–500)	410(262.5–600)	0.10	355 (260–447.5)	380 (250– 450)	0.74
Duration of chest drainage (days, IQR)	2(2–3)	2(2–3)	0.02	2 (2–3)	2 (2–3)	0.13
Length of stay (days, IQR)	3(2–3)	3(3–4)	0.01	3(2–3)	3(3–4)	0.04
Morbidity	2 (4%)	3 (3%)	0.83	1 (2%)	1 (2%)	1.00
Reinsertion of chest drain	0	1	0.46	0	0	1.00
Reoperation	0	0	1.00	0	0	1.00
Mortality	0	0	1.00	0	0	1.00
Lobe						
RUL	21	28		18	17	
RML	3	7		3	3	
RLL	10	14		8	5	
LUL	8	22		6	13	
LLL	8	20		7	4	
pT stage			0.48			0.63
Tis	4	2		3	2	
1a	4	8		2	3	
1b	24	47		22	28	
1c	13	17		10	7	
2a	4	12		4	1	
2b	0	2		0	0	
3	1	3		1	1	
4	0	0		0	0	
Clinical TNM stage			0.21			0.76
IA1	8	4		6	3	
IA2	26	48		22	25	
IA3	11	29		9	11	
IB	2	5		2	2	
IIA	1	3		1	1	
IIB	1	2		1	0	
IIIA	1	0		1	0	
Pathology			0.15			0.09
Adenocarcinoma	43	85		37	41	
Squamous	7	6		5	1	
Solid (*n*, %)	22 (44%)	34 (37%)	0.44	18(43%)	15 (35%)	0.50
Length of tumor (cm, IQR)	1.60 (1.20–2.45)	1.80 (1.20–2.35)	0.47	1.7 (1.2– 2.5)	1.5 (1.2–1.9)	0.18
Lymphadenectomy	10 (6–13)	9 (7–13)	0.29	10 (6.75–13)	8 (6–11)	0.04

*IQR* interquartile range, *FEV1* forced expiratory volume in 1 s, *RUL* right upper lobe, *RML* right middle lobe, *RLL* right lower lobe, *LUL* left upper lobe, *LLL* left lower lobe

### Post-discharge Physical Index

Daily steps (week 1: *p* = 0.094, week 2: *p* = 0.15, week 3: *p* = 0.83) and sleep duration (week 1: *p* = 0.28, week 2: *p* = 0.33, week 3: *p* = 0.98) after discharge also had no statistical difference between the two groups.

### EORTC QLQ-C30 Results

Analyzing the EORTC QLQ-C30 results, in the first week, physical functioning (*p* = 0.75), emotional functioning (*p* = 0.66), cognitive functioning (*p* = 0.94), social functioning (*p* = 0.24), fatigue (*p* = 0.44), nausea and vomiting (*p* = 0.77), dyspnea (*p* = 0.66), insomnia (*p* = 0.39), appetite loss (*p* = 0.47), constipation (*p* = 0.32), diarrhea (*p* = 0.68), and financial difficulties (*p* = 0.71) did not differ significantly between the two groups (Tables 2, 3, 4). However, role functioning (38.49 vs. 53.97, *p* < 0.01), pain (62.30 vs. 45.24, *p* < 0.01), and global health status (43.25 vs. 52.78, *p* < 0.01) of patients in the RATS group were worse than those in the UVATS group. In the second week, all the indicators have no statistical differences, which also means that the role functioning, pain, and global health status of the RATS group were improved. In the third week, there was a shift in the data, and global health status (67.46 vs. 56.15, *p* = 0.03), appetite loss (16.67 vs. 26.98, *p* = 0.04), and diarrhea (7.14 vs. 15.08, *p* = 0.04) scores of the RATS group were better than the UVATS groups. The rest of the data did not show an obvious difference.

**Table 2:** EORTC QLQ-C30 data for first week after discharge

	After propensity score-matched		
	RATS (*n* = 42)	UVATS (*n* = 42)	*p*-value
Function subscale (mean)			
Physical functioning	59.21	57.94	0.75
Role functioning	38.49	53.97	< 0.01
Emotional functioning	71.03	68.85	0.66
Cognitive functioning	78.97	79.37	0.94
Social functioning	64.68	71.83	0.24
Symptoms subscale (mean)			
Fatigue	56.88	52.91	0.44
Nausea and vomiting	13.49	14.68	0.77
Pain	62.30	45.24	< 0.01
Dyspnea	54.76	51.59	0.66
Insomnia	40.48	46.83	0.39
Appetite loss	37.30	32.54	0.47
Constipation	29.37	23.02	0.32
Diarrhea	15.08	17.46	0.68
Financial difficulties	73.02	75.40	0.71
Global health status (mean)			
	43.25	52.78	< 0.01

**Table 3:** EORTC QLQ-C30 data for second week after discharge

	After propensity score-matched		
	RATS (*n* = 42)	UVATS (*n* = 42)	*p*-value
Function subscale (mean)			
Physical functioning	69.37	68.41	0.81
Role functioning	60.71	60.32	0.95
Emotional functioning	75.60	76.39	0.85
Cognitive functioning	82.14	79.76	0.61
Social functioning	67.46	74.60	0.15
Symptoms subscale (mean)			
Fatigue	44.44	43.39	0.83
Nausea and vomiting	6.35	10.71	0.15
Pain	42.46	34.13	0.11
Dyspnea	41.27	40.48	0.89
Insomnia	35.71	34.92	0.90
Appetite loss	23.02	25.40	0.65
Constipation	20.63	15.87	0.41
Diarrhea	7.14	11.90	0.24
Financial difficulties	22.22	27.78	0.36
Global health status (mean)			
	52.58	57.34	0.33

**Table 4:** EORTC QLQ-C30 data for third week after discharge

	After propensity score-matched		
	RATS (*n* = 42)	UVATS (*n* = 42)	*p*-value
Function subscale (mean)			
Physical functioning	76.51	74.76	0.68
Role functioning	68.25	70.63	0.63
Emotional functioning	78.77	72.42	0.16
Cognitive functioning	83.33	77.78	0.22
Social functioning	76.98	71.83	0.26
Symptoms subscale (mean)			
Fatigue	36.24	35.45	0.87
Nausea and vomiting	8.33	11.90	0.27
Pain	32.54	32.14	0.94
Dyspnea	31.75	34.13	0.68
Insomnia	25.40	27.78	0.70
Appetite loss	16.67	26.98	0.04
Constipation	15.87	15.08	0.86
Diarrhea	7.14	15.08	0.04
Financial difficulties	19.84	27.78	0.19
Global health status (mean)			
	67.46	56.15	0.03

### Leicester Cough Questionnaire

The Leicester Cough Questionnaire showed that the scores had no significant difference between the two groups in the first and second weeks (Tables 5, 6, 7). In the third week after discharge, the psychological score (*p* = 0.18) and social score (*p* = 0.27) had no significant difference between the two groups. The physical score of the RATS group was higher than the UVATS group (5.11 vs. 4.58 *p* = 0.04).

**Table 5:** Leicester Cough Questionnaire score for first week after discharge

1 week	Propensity score-matched patients		
	RATS (*n* = 42) (mean)	UVATS (*n* = 42) (mean)	*p*-value
Physical	4.30	4.29	0.97
Psychological	4.62	4.89	0.39
Social	4.95	4.91	0.92
Total scores	13.86	14.09	0.80

**Table 6:** Leicester Cough Questionnaire score for second week after discharge

2 weeks	Propensity score-matched patients		
	RATS (*n* = 42) (mean)	UVATS (*n* = 42) (mean)	*p*-value
Physical	4.81	4.73	0.71
Psychological	4.92	5.01	0.76
Social	5.07	5.22	0.59
Total scores	19.58	20.31	0.49

**Table 7:** Leicester Cough Questionnaire score for third week after discharge

3 weeks	Propensity score-matched patients		
	RATS (*n* = 42) (mean)	UVATS (*n* = 42) (mean)	*p*-value
Physical	5.11	4.58	0.04
Psychological	5.27	4.90	0.18
Social	5.42	5.08	0.27
Total scores	21.56	19.49	0.06

### MD Anderson Symptom Inventory

The pain severity degree of the RATS group was higher than the UVATS group (5.02 vs. 4.10, *p* = 0.04) after the first week. In the second week, all the symptoms did not differ significantly between the two groups. After the third week, the degrees of nausea [0 (0–1) vs. 1 (1–2), *p* = 0.02] and lack of appetite [2 (1–3) vs. 3 (0–3), *p* = 0.04] of the UVATS group were higher than that of the RATS group (Table 8).

**Table 8:** MD Anderson Symptom Inventory with propensity score-matched

	RATS	VATS	*p*-value
1 week			
Symptom items			
Pain (mean)	5.02	4.10	0.04
Fatigue (IQR)	4 (3–6)	5 (3–7)	0.89
Nausea (IQR)	1 (0–3)	1 (0–3)	0.98
Disturbed sleep (IQR)	4 (1.75– 6)	2.5 (1–5.25)	0.37
Distress/feeling upset (IQR)	2 (0–5)	2 (1–6)	0.60
Shortness of breath (IQR)	5 (2–6)	4 (2–6.25)	0.89
Difficulty remembering (IQR)	2 (0–4)	2 (1–3.25)	0.31
Lack of appetite (IQR)	3 (1–4)	2 (0.75–5)	0.52
Drowsiness (IQR)	2 (1–4.25)	3.5 (1–5)	0.61
Dry mouth (IQR)	3 (1–5.25)	2 (1–5)	0.26
Sadness (IQR)	2 (0–5.25)	1 (0.75–4)	0.87
Vomiting (IQR)	0 (0–2)	0 (0–2.25)	0.66
Numbness/tingling (IQR)	2 (0–4)	1 (0–3)	0.16
Interference items			
Activity (IQR)	3 (1–5)	3 (1.75–5)	0.66
Mood (IQR)	3 (1–5)	3 (1–5.25)	0.73
Working (including housework, IQR)	4.5 (2–7)	5 (1.75–7.25)	0.88
Relations with other people (IQR)	1.5 (0–4.25)	0.5 (0–4)	0.46
Walking (IQR)	3 (2–4.25)	3 (0–4)	0.21
Enjoyment of life (IQR)	3 (1–4)	3 (1–5)	0.87
2 weeks			
Symptom items			
Pain (mean)	3.26	3.62	0.47
Fatigue (IQR)	3 (2–4)	4 (2–6.5)	0.17
Nausea (IQR)	0 (0–1)	1 (0–3)	0.03
Disturbed sleep (IQR)	2 (0–4.25)	2 (1–4.25)	0.61
Distress/feeling upset (IQR)	2 (1–4)	2 (1–4.25)	0.85
Shortness of breath (IQR)	3 (2–5)	4 (1–5.25)	0.87
Difficulty remembering (IQR)	1 (0–3)	2.5 (1–3)	0.14
Lack of appetite (IQR)	2 (1–3.25)	2 (1–5)	0.31
Drowsiness (IQR)	2 (1–3)	2 (1–5)	0.42
Dry mouth (IQR)	3 (1–5)	2 (1–5)	0.86
Sadness (IQR)	2 (0–4)	2 (1–4)	0.77
Vomiting (IQR)	1 (0–3)	0 (0–2)	0.18
Numbness/tingling (IQR)	2 (0–4.25)	1 (0–3)	0.50
Interference items			
Activity	3 (1–4)	3 (1–5.25)	0.47
Mood	2.5 (0.75–4)	2 (1–4.25)	0.49
Working (including housework)	3 (2–5.25)	4.5 (2–6.25)	0.55
Relations with other people	1 (0–3.25)	1.5 (0–4.25)	0.45
Walking	2 (1–4)	3 (1–5)	0.80
Enjoyment of life	2 (1–4)	2.5 (1–5)	0.66
3 weeks			
Symptom items			
Pain (mean)	2.93	2.55	0.26
Fatigue (IQR)	3 (2–5)	3 (1–5)	0.43
Nausea (IQR)	0 (0–1)	1 (1–2)	0.02
Disturbed sleep (IQR)	2 (1–4)	1 (0–4)	0.19
Distress/feeling upset (IQR)	2 (1–3.25)	1 (0–4.25)	0.43
Shortness of breath (IQR)	3 (2–4)	4 (2–5.25)	0.51
Difficulty remembering (IQR)	2(1–4)	2 (0–4)	0.54
Lack of appetite (IQR)	2 (1–3)	3 (0–3)	0.04
Drowsiness (IQR)	2 (1–3)	1 (0–3)	0.16
Dry mouth (IQR)	2 (1–3)	1 (0–3.25)	0.34
Sadness (IQR)	1.5 (1–3)	1 (0–4)	0.65
Vomiting (IQR)	0 (0–1)	0 (0–1)	0.40
Numbness/tingling (IQR)	2 (1–4)	1 (0–3)	0.10
Interference items			
Activity (IQR)	3 (1–4)	2 (1–4.25)	0.55
Mood (IQR)	2.5 (1–4)	2 (1–5)	0.92
Working (including housework) (IQR)	3 (2–5)	3 (1–4.25)	0.62
Relations with other people (IQR)	2 (1–4)	1.5 (0–4)	0.43
Walking (IQR)	3 (1–4)	2.5 (1–4)	0.44
Enjoyment of life (IQR)	2 (1–4)	2 (0–5)	0.67

*IQR* interquartile range

### Christensen Fatigue Scale

For the Christensen Fatigue Scale, no significant difference between the two groups was observed (week 1: *p* = 0.82, week 2: *p* = 0.75, week 3: *p* = 0.44; Table 9).

**Table 9:** Christensen Fatigue Scale

		Propensity score-matched patients		
		RATS (*n* = 42)	UVATS (*n* = 42)	*p*-value
1 week, median (IQR)		3 (3–6)	3 (3–6)	0.82
2 weeks, median (IQR)		3 (3–5)	3 (2.75–6)	0.75
3 weeks, median (IQR)		3 (3–4)	3 (2.75–4)	0.44

*IQR* interquartile range

## Discussion

Lung cancer is associated with high mortality and high morbidity.^1^ Lobectomy is the standard surgical approach for stages I–II non-small cell lung cancer.^21^ However, the best method remains unclear. RATS and UVATS are the mainstream methods of lobectomy. Jin et al. concluded that RATS and UVATS had similar perioperative results.^6^ Su Yang et al. found that the RATS is associated with less bleeding and more complete lymphadenectomy than UVATS.^22^ However, while both of the above studies research the short-term outcome of patients, neither study has reported a comparison of the impact of 3–4 weeks of rehabilitation between discharge and return to work for RATS versus UVATS.^6^ In our prospective study, we used the rating scale and portable electronic devices to explore and evaluate the true impact of the two surgical methods. We found that RATS is associated with improved QOL in the third week after discharge.

Postoperative pain is an aspect that cannot be ignored. We noticed that in the first week after discharge, RATS caused worse patient pain than UVATS. According to our interview, in the first week, all the symptoms of the two groups differentiated only in pain, which was not only reflected in the EORTC QLQ-C30 but also in the MD Anderson Symptom Inventory. This result is similar to the Novellis et al. findings published in 2021.^23^ In terms of patient daily disturbance items, the two tables also showed differences in the daily activities of patients. We can thus infer that pain indirectly affects the daily activities of patients. We believe that this phenomenon might be due to the intercostal nerve being crushed and injured during RATS.^24^ The swing of the mechanical arm to the ribs as well as intercostal nerve damage cannot be negligible. The strength of the mechanical arm in RATS is greater when compared with the UVATS assistant manual swing thoracoscope. In addition, three-port RATS destroys two rib intercostal muscles, whereas UVATS only destroys one; another reason might be that RATS may cause prolonged postoperative pain. Now, the single port da Vinci SP has been used in clinical practice and postoperative pain may be relieved due to the development of technique progression.^25^ Moreover, the intercostal muscles are respiratory muscles, which have an impact on lung function. How RATS affects lung function is still unknown and should be evaluated in future studies. If the best solution is relieving pain, then we could prescribe NSAID drugs for patients discharged from hospital, but at the same time, the side effects of this drug class, such as gastric mucosal damage, cannot be ignored.

According to the MD Anderson Symptom Inventory in our study, pain, fatigue, and shortness of breath were the main symptoms of patients at 1 week after discharge. The median severity scores of pain and shortness of breath in the RATS groups were more than 5 points (0–10). The median severity scores of fatigue in the UVATS group were more than 5 points (0–10). By the end of the second week after discharge, the above symptoms were significantly improved in both groups with all the median severity scores reduced to less than 5 points. However, no significant difference was observed between the two groups. In addition, the nausea and lack of appetite severity were better in the RATS group. The results indicated that the life quality of three-port RATS is not inferior to UVATS but is even better than UVATS. However, the symptoms could be improved by preoperative intervention. For example, preoperative administration of steroids drugs could reduce postoperative fatigue and pain caused by surgical inflammatory factors, and preoperative exercise of lung function can reduce postoperative shortness of breath.^26–28^

Cough after lobectomy is also a common complication.^29^ Wu et al. reported that lymphadenectomy is strongly associated with a short-term cough.^30^ There are a number of scholars who believe that injury of the vagus nerve and stimulation of the trachea are the main causes of a postoperative cough.^31^ In our study, the number of lymph node dissection in the RATS group was higher than that in the UVATS group, which means that the trachea and vagus nerve are prone to be damaged. However, our follow-up data suggest that the results are contrary. The cough was more severe in the UVATS group than in the RATS group. We attribute these phenomena to the delicate manipulation of the RATS. The RATS vision system makes it easier to distinguish tissue boundaries.^32^

The assembly of the robot is time consuming and may result in prolonged operation time. Nonetheless, our data suggest that the operation time of RATS is less than UVATS. We believe that the RATS provides an amplified, high-definition, three-dimensional (3D) visualization of the surgical field through employment of a stereoendoscope, which could provide 3D sense and distance to the target for surgeons.^33^ This is especially true when separating the blood vessels and trachea in the lobectomy. The robotic arm could readily raise target blood vessels and the trachea, allowing the assistant to quickly sever and resect them with endoscopic staplers. The fully exposing surgical field, the flexible mechanical arm, and clear 3D imaging could help the surgeon complete each operation easily and quickly.^34^ However, it is worth noting that any technique has its learning curve, and robotic surgery is no exception. Increased experience with robotic operations will dramatically reduce operation time,^35^ and shorter anesthetic procedure time would result in the use of fewer anesthesia drugs. Although the incidence of postoperative complications was not statistically significant between the two groups in our study, it has been shown in other studies. Sinclair et al. and Apfel et al. concluded that the longer the anesthesia time and the larger the anesthesia dose, the higher the incidence of postoperative nausea and vomiting (PONV).^36,37^ Kim et al. concluded that an increased risk of venous thromboembolism is associated with an increased duration of anesthesia.^38^

Lymph node dissection is an important procedure for lobectomy, which is related to the postoperative pathological stage and further treatment options. The more lymph nodes examined results in more accurate nodal staging and better long-term survival after resection of non-small cell lung cancer.^39^ However, lymph node resection is associated with a risk of chylothorax and nerve injury.^40^ Our data suggested that more lymph nodes are removed by RATS, but the complications did not increase. This may be due to the flexible mechanical arm of RATS, which could reduce the hand shaking of the surgeon.^41^ Thus, the complex mediastinal lymph node resection under RATS could be easily accomplished.^22,34^ Fine manipulation by RATS could also reduce the tissue damage during surgery,^42^ which would result in a reduction of drainage volume. However, this phenomenon was not observed in our study. Although median drainage volume of the RATS group is lower than the UVATS group, it was not statistically significant. We thought it might be because of more lymph nodes dissected. The advantages of RATS in dissecting lymph nodes might include less damage to lymphatic vessels and their surrounding vessels.^43^ More lymph nodes dissected means more tissue damaged and increased postoperative drainage.^44^ All in all, RATS is more suitable for those who had difficulty with dissection, especially for those who underwent neoadjuvant therapy or had suspected lymph node metastasis. The advantages of RATS lymph node excision suggest a greater advantage in the treatment of lung cancer than UVATS. However, the data in our study did not support the hypothesis. A long-term survival study is requisite.

Although RATS has many advantages, the expense is an issue that cannot be ignored. Cost was not analyzed in our study. In Shandong Provincial Hospital affiliated with Shandong First Medical University, the da Vinci robot costs about $2900 USD once and is not covered by most commercial and government insurance. The cost significantly limits the use of RATS.^45,46^ In our study, the financial difficulty rates in EORTC QLQ-C30 were not statistically significant between the RATS and UVATS groups, because the choice of surgical method is the patient’s own decision. The high cost of robots is the reason why we did not perform a randomized control trial.

This study has several limitations. First, in our study, we found patients in the UVATS group had more severe gastrointestinal reactions. However, the reasons for this need further research. Second, although we adopted a propensity score analysis to eliminate bias, the sample size of the enrolled patients was still too small, and the results are not generalizable. Third, it takes about 2 days for patients to complete the necessary examinations before surgery, and the difference between the ward environment and the patient’s home environment makes it difficult to collect accurate baseline data before surgery. Lastly, it is also a pity that we have not yet compared the postoperative pulmonary function. Follow-up pulmonary function testing was scheduled after 6 months but has not yet been completed. Additionally, the short follow-up period of only 3 weeks post-discharge limited the evaluation of long-term convalescence.

## Conclusions

Three-port RATS and uniportal UVATS each have advantages, and RATS is a safe and feasible alternative to VATS for patients with NSCLC. During the 3-week postoperative follow-up, RATS was associated with short-term postoperative pain but less postoperative complications. Three-port RATS was not inferior to uniportal UVATS with two additional portals.
